# Parathyroid carcinoma in a nonorthotopic supraclavicular location: a 20-year course

**DOI:** 10.1210/jcemcr/luag225

**Published:** 2026-09-03

**Authors:** Kimitaka Shibue, Ryo Tsukaguchi, Sachiko Honjo, Akihiro Hamasaki

**Affiliations:** Department of Diabetes and Endocrinology, Medical Research Institute KITANO HOSPITAL, PIIF Tazuke-Kofukai, Kita-ku, Osaka 530-8480, Japan; Department of Diabetes and Endocrinology, Medical Research Institute KITANO HOSPITAL, PIIF Tazuke-Kofukai, Kita-ku, Osaka 530-8480, Japan; Department of Diabetes and Endocrinology, Medical Research Institute KITANO HOSPITAL, PIIF Tazuke-Kofukai, Kita-ku, Osaka 530-8480, Japan; Department of Diabetes and Endocrinology, Medical Research Institute KITANO HOSPITAL, PIIF Tazuke-Kofukai, Kita-ku, Osaka 530-8480, Japan

**Keywords:** primary hyperparathyroidism, parathyroid carcinoma, hypercalcemia, recurrent parathyroid disease, multigland parathyroid surgery, nonorthotopic parathyroid lesion

## Abstract

Parathyroid carcinoma is a rare cause of primary hyperparathyroidism (PHPT) and is often difficult to distinguish from benign parathyroid disease in its early stages. We describe a nearly 20-year clinical course of recurrent PHPT requiring 4 surgical interventions, ultimately culminating in the diagnosis of parathyroid carcinoma in a nonorthotopic supraclavicular location. The patient was followed continuously at our institution, allowing longitudinal assessment of biochemical, imaging, and histopathological findings. The initial surgery resulted in biochemical remission following removal of a parathyroid adenoma; however, hypercalcemia and elevated intact parathyroid hormone levels recurred over time. Subsequent resections demonstrated parathyroid-type proliferation with increasing Ki-67 index, and the final surgery revealed capsular and venous invasion consistent with carcinoma. Notably, technetium-99 m methoxyisobutylisonitrile uptake became progressively weaker over time and eventually appeared as faint uptake in a nonorthotopic location. These findings may reflect changes in tumor biology over time. Although it remains uncertain whether the supraclavicular lesion represented an ectopic primary carcinoma or metastatic disease from an occult orthotopic lesion, this case highlights the diagnostic challenges of recurrent PHPT and the importance of long-term follow-up, reassessment of atypical locations, and careful interpretation of changing imaging and proliferative indices.

## Introduction

Parathyroid carcinoma is a rare endocrine malignancy accounting for <1% of primary hyperparathyroidism (PHPT) cases. Its clinical presentation frequently overlaps with benign adenoma, as early manifestations may include only modest hypercalcemia or mild elevation of parathyroid hormone (PTH), and histopathology from initial surgeries may lack definitive malignant features. Accordingly, recurrent or persistent hypercalcemia following parathyroidectomy should prompt consideration of malignancy [1-3]. Parathyroid carcinoma may occur sporadically or in association with germline pathogenic variants, most commonly involving *CDC73*, which is linked to hyperparathyroidism–jaw tumor syndrome [4]. Epidemiologic estimates indicate an incidence of roughly 11 cases per 10 million individuals, underscoring both its rarity and the difficulty of early recognition [5]. Ectopic parathyroid glands occur in ∼6-16% of patients with PHPT, most commonly in the thymus, retroesophageal region, or within the thyroid gland; however, parathyroid carcinoma arising from ectopic tissue is exceedingly rare [6, 7].

We describe a 20-year clinical course of PHPT requiring 4 surgeries, ultimately revealing carcinoma within an ectopic supraclavicular parathyroid gland. This case highlights the diagnostic complexity of recurrent PHPT, evolving histopathology, and the importance of reassessing ectopic disease when hypercalcemia persists despite previous surgeries. In addition, this case demonstrated progressive changes in 99mTc-methoxyisobutylisonitrile uptake over time, which may reflect changes in tumor cell composition and mitochondrial content, providing insight into the biological behavior of parathyroid tumors.

## Case presentation

A male patient in his 70s was diagnosed with PHPT following routine detection of hypercalcemia and elevated intact PTH. There was no family history suggestive of multiple endocrine neoplasia type 1 (MEN-1). MEN-1 was clinically excluded based on the absence of pancreatic or pituitary lesions and normal catecholamine and pituitary hormone levels. He was continuously followed at the authors' institution thereafter. Detailed perioperative biochemical data before and after each of the 4 surgical procedures are summarized in Table 1.

**Table 1:** Biochemical data before and after each of the 4 surgical procedures

Surgery	Time point	Serum calcium, mg/dL (SI: mmol/L)	Intact PTH, pg/mL (SI: pmol/L)	Interpretation
Px1	POD –53	11.4 mg/dL (SI: 2.85 mmol/L)	289 pg/mL (SI: 30.6 pmol/L)	Biochemical remission
Px1	POD 1	8.5 mg/dL (SI: 2.13 mmol/L)	18 pg/mL (SI: 1.89 pmol/L)	
Px1	POD 4	8.4 mg/dL (SI: 2.10 mmol/L)	87 pg/mL (SI: 9.16 pmol/L)	
Px2	POD –26	11.4 mg/dL (SI: 2.85 mmol/L)	289 pg/mL (SI: 30.6 pmol/L)	Persistent disease
Px2	POD 2	8.5 mg/dL (SI: 2.13 mmol/L)	18 pg/mL (SI: 1.89 pmol/L)	
Px3	POD 0, before surgery	8.9 mg/dL (SI: 2.23 mmol/L)	1990 pg/mL (SI: 211 pmol/L)	Persistent disease
Px3	POD 0, after surgery	8.4 mg/dL (SI: 2.10 mmol/L)	226 pg/mL (SI: 23.9 pmol/L)	
Px4	POD –2	10.7 mg/dL (SI: 2.68 mmol/L)	1905 pg/mL (SI: 202 pmol/L)	Temporary biochemical remission
Px4	POD 2	9.2 mg/dL (SI: 2.30 mmol/L)	202 pg/mL (SI: 21.4 pmol/L)	

Detailed biochemical data before and after each of the 4 surgical procedures are shown. Reference ranges are provided below. Reference ranges: serum calcium, 8.5-10.1 mg/dL (SI: 2.10-2.60 mmol/L); intact PTH, 10-65 pg/mL (SI: 1.05-6.84 pmol/L).

Abbreviations: iPTH, intact parathyroid hormone; POD, postoperative day; PTH, parathyroid hormone; Px, sequential surgical procedure.

First surgery (Px1): One year after diagnosis, bilateral lower parathyroid exploration was performed. A 3-mm nodule near the left lower pole was excised, and a right lower lesion was confirmed as a parathyroid adenoma. The small left lower nodule was not considered to be the primary cause of hyperparathyroidism based on intraoperative findings and postoperative biochemical remission after removal of the right lower adenoma. Histopathology showed cuboidal cells with mild atypia in solid and trabecular arrangements, surrounded by a fibrous capsule, without features of carcinoma. Following the first surgery, serum calcium and intact PTH levels normalized, indicating biochemical remission. Postoperatively, calcium lactate at 9 g/day and alfacalcidol at 1 mcg/day were administered temporarily.

**Figure 1:**
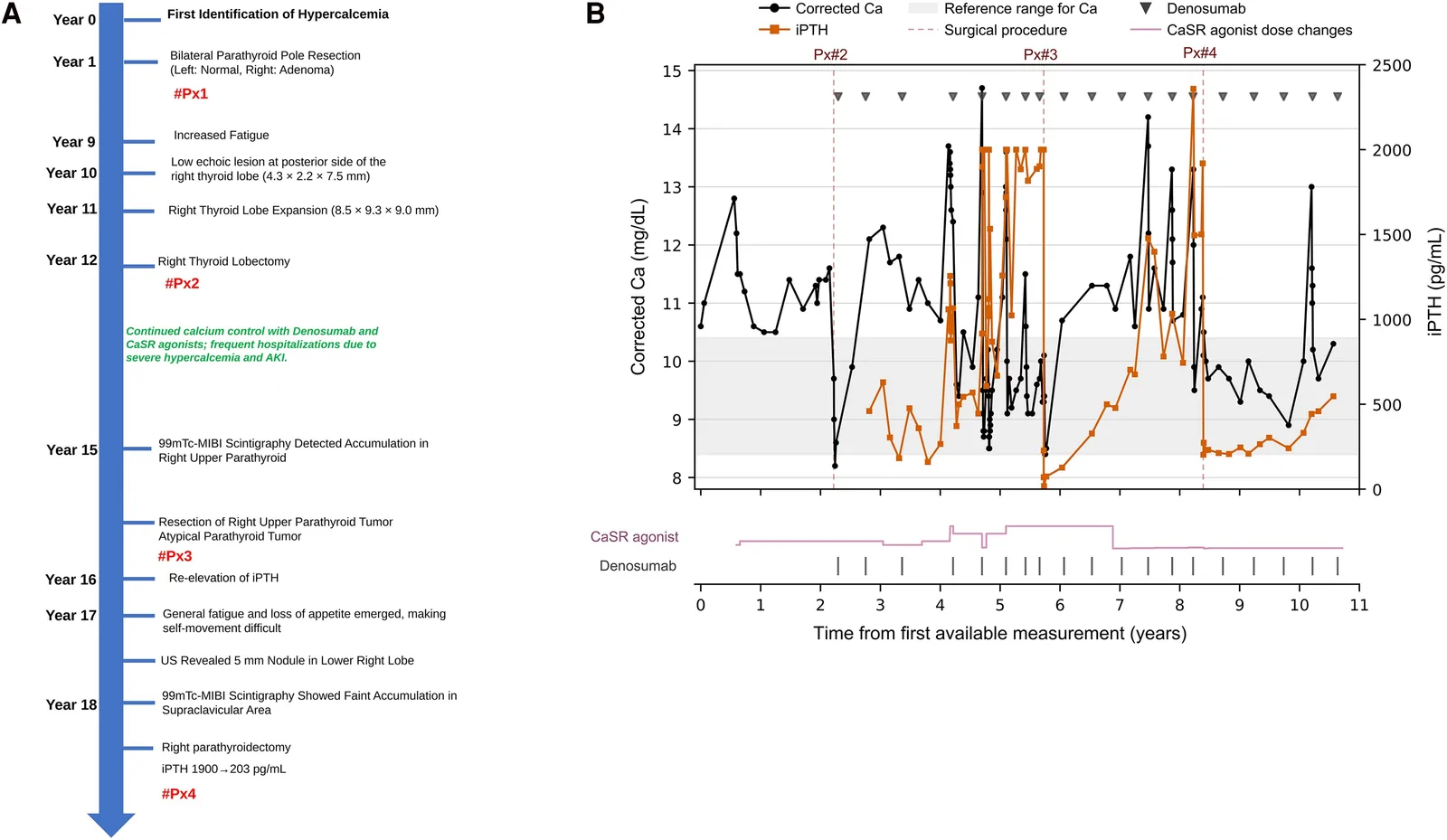
Overview of the clinical course and longitudinal changes in serum calcium and intact parathyroid hormone (iPTH) levels in relation to surgical interventions and medical treatment. A) Overview of the patient's clinical course since diagnosis of primary hyperparathyroidism. B) Time course of serum calcium and iPTH levels in relation to surgical procedures and medical treatments. Serum calcium and iPTH values shown immediately before each surgical procedure represent preoperative values, whereas values shown after each procedure represent postoperative measurements obtained during follow-up. The graphs show serum calcium, iPTH (upper panel), and the dosing histories of cinacalcet and evocalcet (lower panel), plotted against time from the first available measurement. Vertical dashed lines indicate the timing of the subsequent surgical procedures: right thyroid lobectomy at Px2, resection of an atypical parathyroid tumor at Px3, and resection of parathyroid carcinoma at Px4. Downward-pointing triangles indicate denosumab administration. Drug doses were adjusted according to serum calcium levels.

Second surgery (Px2): Nine years after the initial diagnosis, biochemical recurrence of PHPT was suspected due to progressive increases in serum calcium and intact PTH. Imaging demonstrated gradual enlargement of a lesion in the caudal right thyroid lobe, and ultrasonography identified a 4.3 × 2.2 × 7.5 mm hypoechoic nodule (Fig. 1A). A second surgical exploration was undertaken, and several nodules associated with the right thyroid lobe were removed, necessitating lobectomy. Histopathology showed adenomatous goiter, indicating that the resected lesion was thyroid tissue rather than parathyroid tissue. Serum intact PTH and calcium showed a transient postoperative decline coinciding with intermittent denosumab administration at a dose of 60 mg per dose, followed by re-elevation (Fig. 1B), indicating persistent PHPT.

**Figure 2:**
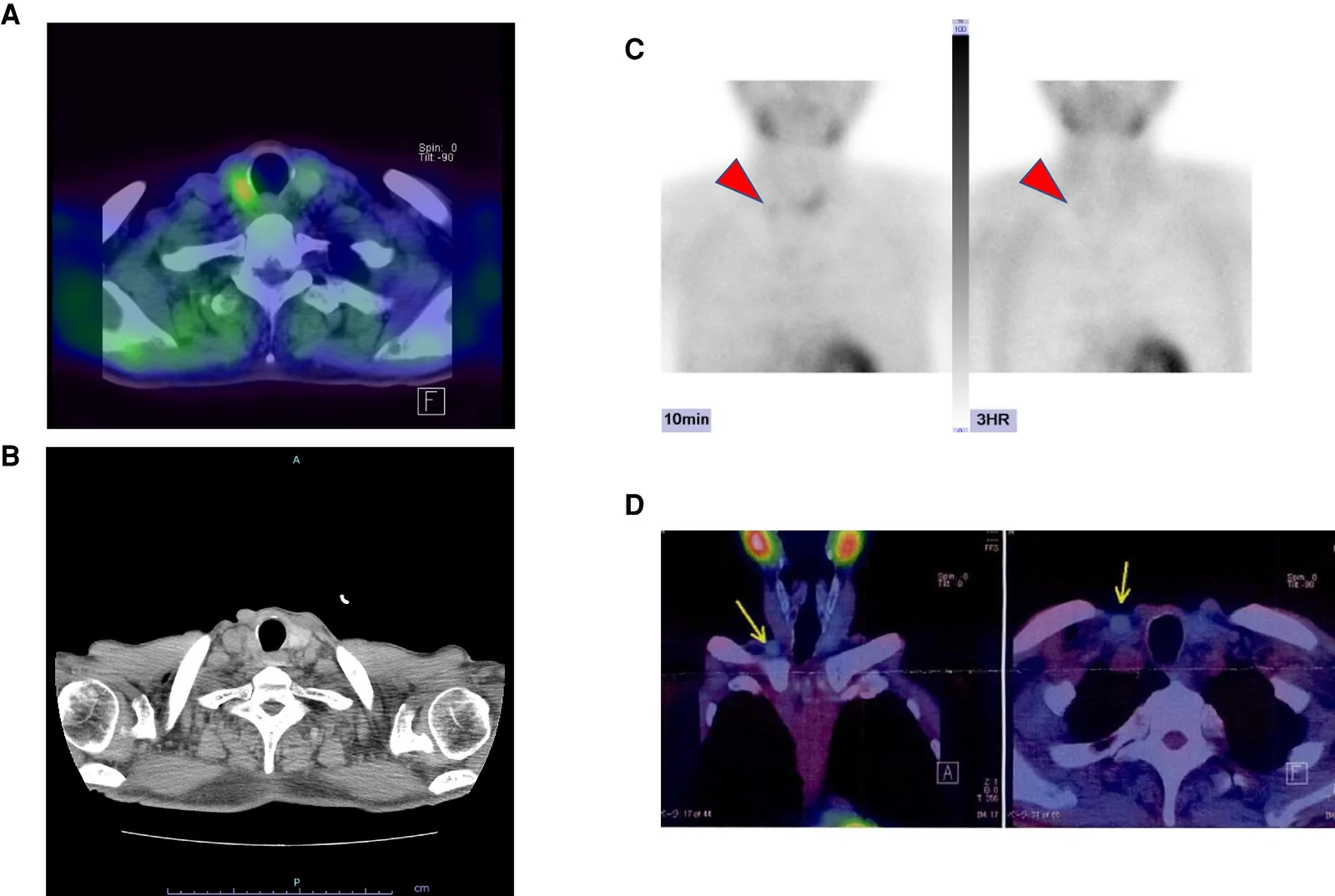
Imaging findings during the workups for the third and fourth surgical procedures (Px3 and Px4). A and B) A coronal fused single-photon emission computed tomography/computed tomography (SPECT/CT) image (A) and a CT scan (B) obtained before Px3, 15 years after the initial diagnosis of hypercalcemia. Arrows indicate the site of focal 99mTc-MIBI accumulation in (A) and the corresponding anatomical lesion on CT in (B). C and D) Imaging findings obtained during the workup for Px4, 18 years after the initial diagnosis, include a 99mTc-MIBI scan (C) showing increased tracer uptake in the right supraclavicular region (arrows) and a coronal fused SPECT/CT image (D) showing faint abnormal 99mTc-MIBI uptake in the right supraclavicular region (arrow).

Third surgery (Px3): Fifteen years after the initial diagnosis, the intact PTH level exceeded 1990 pg/mL (SI: 211 pmol/L) (reference range, 10-65 pg/mL [SI: 1.05-6.84 pmol/L]) (Fig. 1A and 1B). Technetium-99 m methoxyisobutylisonitrile (99mTc-MIBI) scintigraphy performed prior to the third surgery demonstrated focal uptake in the right upper parathyroid region (Fig. 2A and 2B), which guided the surgical exploration. Re-exploration revealed extensive fibrosis. A mass in the tracheoesophageal groove was excised en bloc with sacrifice of the recurrent laryngeal nerve, along with adjacent reddish nodules. Histopathology showed parathyroid-type proliferation with uniform nuclei and fibrous septa, and Ki-67 positivity of ∼10%. Definitive capsular or vascular invasion was not identified, precluding a diagnosis of carcinoma. Retrospectively, these findings are best interpreted as an atypical parathyroid tumor according to the 2022 World Health Organization (WHO) classification.

Seventeen years after diagnosis, the patient was rehospitalized for severe hypercalcemia, with a corrected serum calcium concentration of 14.2 mg/dL (SI: 3.55 mmol/L) (reference range, 8.5-10.1 mg/dL [SI: 2.10-2.60 mmol/L]). Although the level briefly improved with treatment, hypercalcemia later recurred.

## Diagnostic assessment

At 18 years after diagnosis, the serum calcium concentration reached 13.3 mg/dL (SI: 3.32 mmol/L), and the intact PTH level rose to 2358 pg/mL (SI: 250 pmol/L). Ultrasound and computed tomography (CT) showed no cervical abnormalities. Repeat 99mTc-MIBI scintigraphy performed prior to the fourth surgery identified a new faint focus of uptake in the right supraclavicular fossa (Fig. 2C and 2D), which led to surgical exploration of this region. This suggested progressive changes in tumor cell composition. Differential diagnoses included recurrent adenoma, multigland hyperplasia, parathyroid carcinoma, ectopic vs metastatic parathyroid tissue, and thyroid neoplasm. Fine-needle aspiration was avoided because cytology is not reliable for distinguishing benign from malignant parathyroid lesions, and biopsy is generally not recommended when parathyroid carcinoma is suspected because of the risk of local implantation or needle-tract seeding [8, 9].

## Treatment

**Figure 3:**
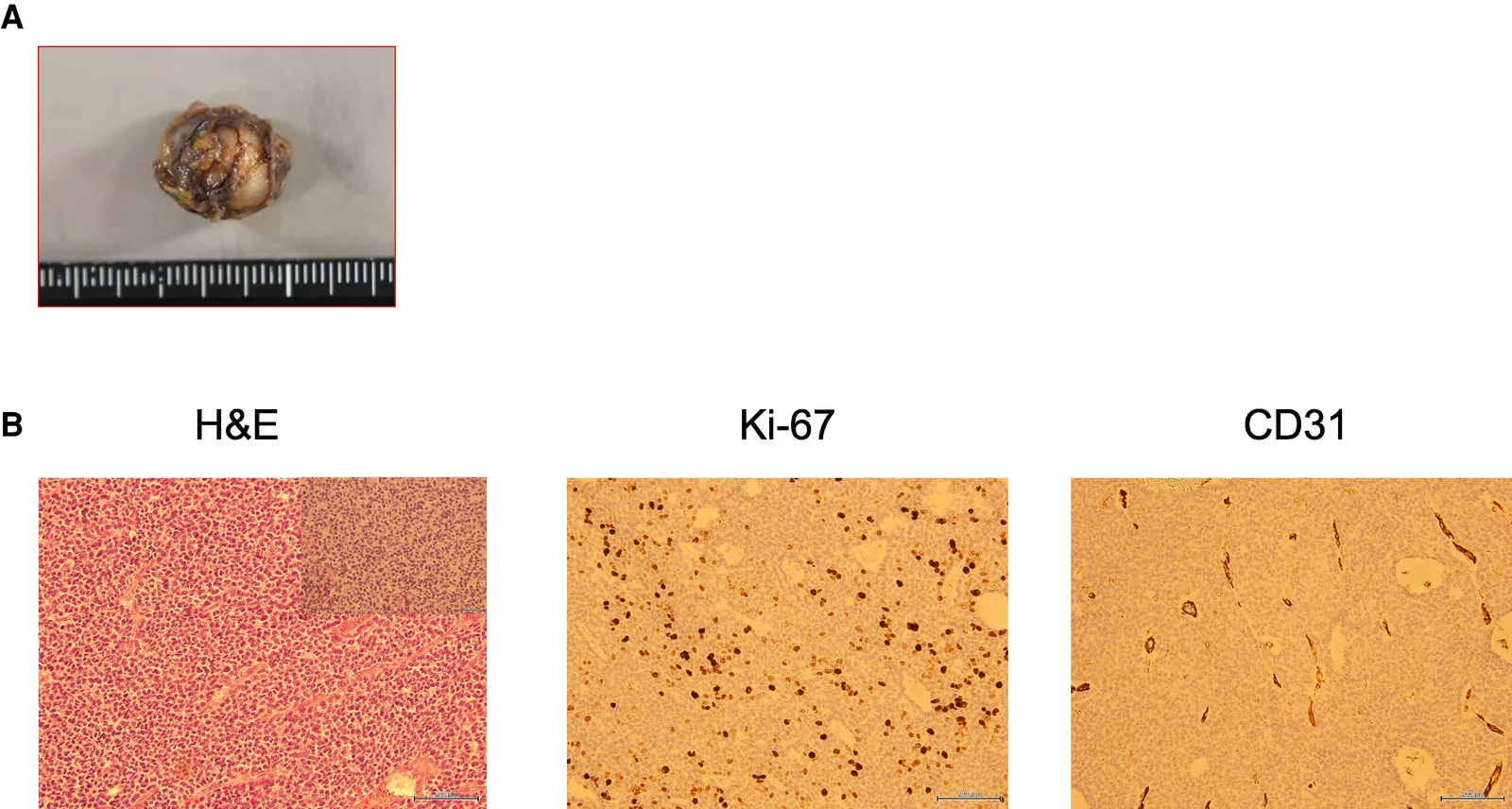
Pathological findings of the excised tumor at the fourth surgery (Px4). A and B) A gross image of the excised tumor (A) is provided, alongside histopathological images of the excised tumor (B), which display hematoxylin and eosin (H&E) staining, Ki-67 staining, and CD31 staining. Objective magnification: ×20. Scale bar, 200 μm. Insets show digitally enlarged views of the corresponding images.

Fourth surgery (Px4): A 2.5-cm supraclavicular incision exposed a well-circumscribed mass. The tumor was dissected subcapsularly to avoid seeding; it was loosely adherent to the internal jugular vein and exhibited medial scarring. Histopathology revealed solid proliferation of parathyroid-type cells with internal fibrous septa, a Ki-67 index of 20-30%, and multifocal capsular and venous invasion confirmed by elastica van Gieson and CD31 staining (Fig. 3A and 3B), establishing the diagnosis of parathyroid carcinoma in a nonorthotopic supraclavicular region. The tumor was removed en bloc with surrounding soft tissue, and the surgical margin was considered macroscopically negative. No enlarged lymph nodes were identified, and lymph node sampling was not performed.

**Figure 4:**
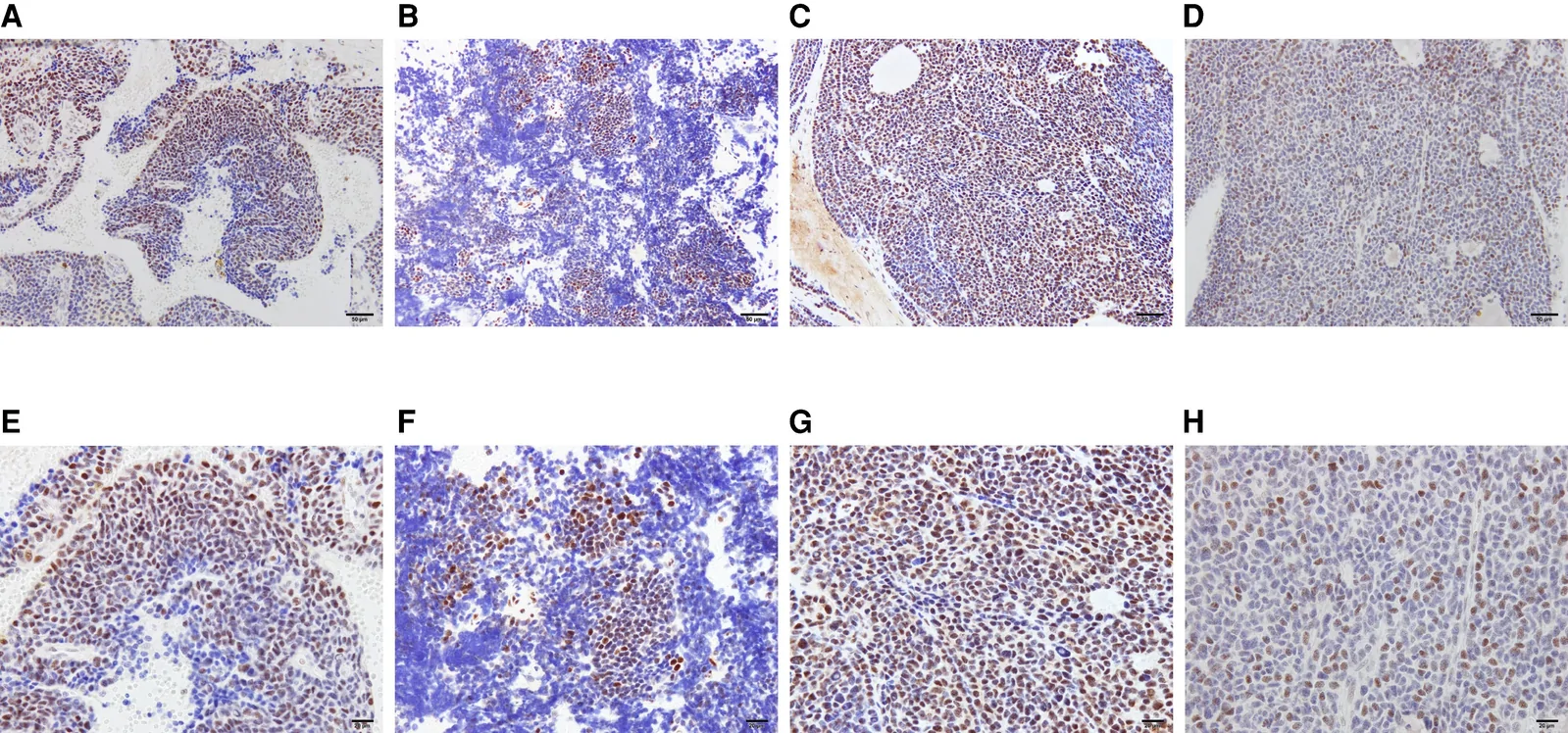
Retrospective parafibromin immunohistochemistry of specimens from the 4 surgeries. Parafibromin immunohistochemistry was performed retrospectively on available specimens from all 4 surgeries. Low-power images are shown for the first parathyroidectomy (Px1) (A), second surgery (Px2) (B), third surgery (Px3) (C), and fourth surgery (Px4) (D). Corresponding high-power images are shown for Px1 (E), Px2 (F), Px3 (G), and Px4 (H). Nuclear parafibromin expression was retained in evaluable cells in all examined specimens, including the parathyroid adenoma from Px1, the thyroid tissue removed at Px2, the atypical parathyroid tumor from Px3, and the parathyroid carcinoma from Px4. Loss of nuclear parafibromin expression was not identified in any of the examined specimens. Objective magnification: A–D, ×20; E–H, ×40. Scale bars: A–D, 50 μm; E–H, 20 μm.

To further evaluate possible *CDC73*-related disease biology, retrospective parafibromin immunohistochemistry was performed on available specimens from all 4 surgeries in consultation with the pathologist. Parafibromin immunohistochemistry was performed using a prediluted ready-to-use mouse monoclonal anti-parafibromin antibody, clone BSB-50 (catalog no. BSB 2747; Bio SB, Santa Barbara, CA, USA), according to the institutional pathology laboratory protocol. Other histochemical and immunohistochemical stains were performed as part of routine diagnostic pathology practice. Nuclear parafibromin expression was retained in the parathyroid lesions from the first, third, and fourth surgeries, including the carcinoma specimen from the fourth surgery. The thyroid tissue removed at the second surgery also showed retained nuclear parafibromin expression in evaluable cells. Thus, loss of nuclear parafibromin expression was not identified in any of the examined surgical specimens (Fig. 4).

## Outcome and follow-up

After Px4, serum calcium and intact PTH decreased and temporarily stabilized. However, intact PTH remained mildly elevated during follow-up despite initial improvement in calcium levels, which may reflect microscopic residual disease or the known tendency for parathyroid carcinoma to recur even after apparently complete resection. Maintenance therapy with evocalcet was restarted at 2 mg/day, titrated to 4 mg/day and then to 8 mg/day, and subsequently continued at 8 mg/day. Intermittent denosumab administration at a dose of 60 mg per dose was also continued.

Over the following 3 years, mild biochemical recurrence was noted, although 18F-fluorodeoxyglucose positron emission tomography/computed tomography (18F-FDG PET/CT) showed no metastasis. Owing to the propensity for late recurrence in parathyroid carcinoma, extended surveillance was maintained. A summary of the 4 surgical interventions, lesion locations, histopathological diagnoses, and representative perioperative biochemical findings is provided in Table 2.

**Table 2:** Summary of the 4 surgical interventions, lesion locations, histopathological diagnoses, parafibromin immunohistochemistry, and representative biochemical findings

Surgery	Year from diagnosis	Location of lesion	Surgical procedure	Histopathological diagnosis	Parafibromin immunohistochemistry	Preoperative serum calcium, mg/dL (SI: mmol/L)	Preoperative intact PTH, pg/mL (SI: pmol/L)	Early postoperative serum calcium, mg/dL (SI: mmol/L)	Early postoperative intact PTH, pg/mL (SI: pmol/L)	Interpretation
Px1	1 year	Right lower parathyroid	Parathyroidectomy	Parathyroid adenoma	Retained nuclear expression	11.4 mg/dL (SI: 2.85 mmol/L)	289 pg/mL (SI: 30.6 pmol/L)	8.5 mg/dL (SI: 2.13 mmol/L)	18 pg/mL (SI: 1.89 pmol/L)	Biochemical remission
Px2	9 years	Right thyroid lower pole	Right thyroid lobectomy	Adenomatous goiter	Retained nuclear expression in evaluable thyroid tissue	11.4 mg/dL (SI: 2.85 mmol/L)	289 pg/mL (SI: 30.6 pmol/L)	8.5 mg/dL (SI: 2.13 mmol/L)	18 pg/mL (SI: 1.89 pmol/L)	Persistent disease despite transient biochemical improvement
Px3	15 years	Right upper parathyroid region/tracheoesophageal groove	Tumor resection	Atypical parathyroid tumor	Retained nuclear expression	8.9 mg/dL (SI: 2.23 mmol/L)	1990 pg/mL (SI: 211 pmol/L)	8.4 mg/dL (SI: 2.10 mmol/L)	226 pg/mL (SI: 23.9 pmol/L)	Persistent disease
Px4	18 years	Right supraclavicular region	Tumor resection	Parathyroid carcinoma	Retained nuclear expression	10.7 mg/dL (SI: 2.68 mmol/L)	1905 pg/mL (SI: 202 pmol/L)	9.2 mg/dL (SI: 2.30 mmol/L)	202 pg/mL (SI: 21.4 pmol/L)	Temporary biochemical remission

This table summarizes the lesion site, surgical procedure, histopathological diagnosis, parafibromin immunohistochemistry, and representative perioperative biochemical values for each of the 4 surgeries. Preoperative and early postoperative serum calcium and intact PTH values are shown to illustrate the biochemical course across the patient's long-term clinical follow-up. Parafibromin immunohistochemistry was performed retrospectively on available specimens from all 4 surgeries. Retained nuclear expression indicates that loss of nuclear parafibromin staining was not identified in the examined specimen. Reference ranges: serum calcium, 8.5-10.1 mg/dL (SI: 2.10-2.60 mmol/L); intact PTH, 10-65 pg/mL (SI: 1.05-6.84 pmol/L).

Abbreviations: iPTH, intact parathyroid hormone; PTH, parathyroid hormone; Px, sequential surgical procedure.

## Discussion

Parathyroid carcinoma is a rare endocrine malignancy that is often difficult to distinguish from benign parathyroid disease in its early stages because clinical, biochemical, and histopathological findings may overlap [1-3]. In this case, the patient underwent multiple surgeries over nearly 2 decades before a definitive diagnosis of carcinoma was established. The prolonged clinical course illustrates the diagnostic challenges associated with recurrent or persistent PHPT.

An important clinical question in this case is whether the initial lesion represented benign adenoma and whether the subsequent lesions represented recurrent benign disease, atypical parathyroid tumor, or carcinoma. The first operation resulted in normalization of serum calcium and an intact PTH levels, suggesting that the right lower parathyroid adenoma removed at that time was clinically functional and responsible for PHPT at the initial presentation. However, recurrent hypercalcemia and progressive elevation of intact PTH developed over time, ultimately leading to the diagnosis of carcinoma at the fourth surgery.

Reinterpretation of the histopathological findings from earlier surgeries using the current WHO classification is informative. The lesion removed at the third surgery demonstrated parathyroid-type proliferation with fibrous septa and a Ki-67 index of ∼10%, without definitive capsular or vascular invasion. Retrospectively, these features are best interpreted as an atypical parathyroid tumor rather than a typical adenoma. Atypical parathyroid tumor is an intermediate category for parathyroid tumors showing atypical histological features that do not fulfill the diagnostic criteria for carcinoma, such as unequivocal capsular or vascular invasion.

In this patient, the Ki-67 index increased over successive resections, from low proliferative activity in the initial lesion to ∼10% at the third surgery and 20-30% at the fourth surgery. Reported Ki-67 indices are typically <1-2% in adenomas, ∼3-5% in atypical parathyroid tumors, and often >5-10% in carcinomas [10]. The stepwise increase in proliferative activity in this case may suggest a gradual change in tumor biology over time, although definitive malignant transformation cannot be proven from a single case. It is also possible that a low-grade carcinoma had been present but not recognized in earlier stages, given the known histological overlap between atypical parathyroid tumor and carcinoma. Clinically, the diagnosis of atypical parathyroid tumor is important because it is associated with a higher risk of recurrence than typical adenoma and therefore requires careful long-term biochemical and imaging follow-up. The distinction between atypical parathyroid tumor and low-grade carcinoma is known to be challenging, and some tumors initially classified as atypical parathyroid tumor may later demonstrate malignant behavior [11, 12].

Another notable feature in this case was the change in 99mTc-methoxyisobutylisonitrile (MIBI) uptake over time. Parathyroid adenomas often demonstrate strong MIBI uptake due to abundant mitochondria in oxyphil cells, whereas parathyroid carcinomas may contain a higher proportion of chief cells with lower mitochondrial content, resulting in weaker tracer uptake [13-16]. In this patient, MIBI uptake became progressively weaker and eventually appeared as faint uptake in a nonorthotopic location, which may reflect changes in tumor cell composition and mitochondrial content over time. This finding suggests that faint MIBI uptake does not exclude carcinoma, particularly in patients with long-standing or recurrent disease.

The tumor identified at the fourth surgery was in the supraclavicular region, which is not a typical orthotopic location for parathyroid glands. Although ectopic parathyroid glands are found in ∼6-16% of patients with PHPT [6, 7], parathyroid carcinoma accounts for <1% of PHPT cases [1-3]. Therefore, parathyroid carcinoma arising in an ectopic or nonorthotopic location is extremely rare. In this case, the supraclavicular tumor was interpreted as parathyroid carcinoma arising in a nonorthotopic location. However, it is difficult to definitively distinguish ectopic primary carcinoma from metastatic disease arising from a previously unrecognized orthotopic primary tumor. The solitary nature of the lesion, absence of distant metastasis, and the long clinical course without evidence of widespread disease may support an ectopic origin, but metastatic disease cannot be completely excluded. Therefore, this case should be interpreted as parathyroid carcinoma identified in a nonorthotopic supraclavicular location after a prolonged clinical course of recurrent PHPT, rather than definitive proof of de novo carcinoma arising from ectopic tissue.

Genetic factors such as germline pathogenic variants in *CDC73* are associated with parathyroid carcinoma and hyperparathyroidism–jaw tumor syndrome [4, 12]. Because *CDC73* status was central to the diagnostic interpretation in this case, we performed retrospective parafibromin immunohistochemistry on the available surgical specimens from all 4 operations. Nuclear parafibromin expression was retained in the examined lesions, including the carcinoma diagnosed at the fourth surgery. Therefore, the immunohistochemical findings did not provide evidence of loss of parafibromin expression or support a *CDC73*-inactivated tumor phenotype.

This result is clinically informative but should be interpreted cautiously. Retained parafibromin expression does not exclude parathyroid carcinoma, and the diagnosis in the fourth specimen was based on definitive histopathological evidence of multifocal capsular and venous invasion rather than on parafibromin loss. In addition, because all examined specimens retained nuclear parafibromin expression, parafibromin immunohistochemistry could not determine whether the recurrent lesions represented a single clonal process, separate metachronous tumors, ectopic primary carcinoma, or metastatic disease from an occult orthotopic lesion. Germline and somatic *CDC73* sequencing were not performed, which remains a limitation of this report. Nevertheless, the retained parafibromin expression across all specimens argues against, but does not definitively exclude, a *CDC73*-inactivated malignant spectrum underlying the entire 20-year course.

This case highlights several important clinical lessons. First, recurrent or persistent PHPT after parathyroidectomy should prompt long-term biochemical and imaging follow-up, as recurrence may occur many years after initial surgery. Second, atypical parathyroid tumors with elevated proliferative indices may require careful surveillance due to the potential for recurrence. Third, when orthotopic parathyroid glands appear normal, ectopic or nonorthotopic locations should be carefully evaluated. Finally, changes in imaging characteristics, such as decreasing MIBI uptake, may reflect changes in tumor biology and should not be interpreted as excluding malignancy.

In conclusion, this case illustrates the diagnostic challenges of recurrent PHPT over a prolonged clinical course. The increasing Ki-67 index and evolving imaging findings suggest a stepwise change in tumor biology over time. Although it remains uncertain whether the supraclavicular tumor represented an ectopic primary carcinoma or metastatic disease from an occult orthotopic lesion, this case emphasizes the importance of long-term follow-up and repeated evaluation, including assessment of nonorthotopic locations, in patients with persistent or recurrent hyperparathyroidism.

## Learning points

- Parathyroid carcinoma may be difficult to distinguish from benign or atypical parathyroid tumors in the early stages, and recurrent PHPT requires long-term follow-up.
- Atypical parathyroid tumors with elevated proliferative indices may recur and require careful surveillance.
- Parathyroid lesions may arise in nonorthotopic or ectopic locations, and these areas should be evaluated when hyperparathyroidism persists despite previous surgery.
- Decreasing 99mTc-methoxyisobutylisonitrile uptake over time may reflect changes in tumor cell composition and does not exclude malignancy.
- The distinction between atypical parathyroid tumor and carcinoma may be difficult, and long-term clinical follow-up is essential in patients with recurrent or persistent disease.

## Contributors

All authors made individual contributions to authorship. K.S. and R.T. were directly involved in the clinical care of the patient. K.S. collected and interpreted the clinical data, acquired the clinical and histopathological images, and drafted the manuscript. R.T. contributed to the interpretation of the clinical course. S.H. and A.H. provided overall supervision and critically revised the manuscript. All authors approved the final version of the manuscript and agree to be accountable for the work.

## Data Availability

Original data generated and analyzed for this case report are included in this published article.
